# Expression and immunohistochemical localization of leptin in human periapical granulomas

**DOI:** 10.4317/medoral.20385

**Published:** 2015-02-07

**Authors:** Jénifer Martín-González, Antonio Carmona-Fernández, Antonio Pérez-Pérez, Flora Sánchez-Jiménez, Víctor Sánchez-Margalet, Juan J. Segura-Egea

**Affiliations:** 1 DDS, Doctoral fellow, Department of Endodontics, School of Dentistry, University of Sevilla, Sevilla, Spain; 2 BSc, PhD, Department of Medical Biochemistry, Molecular Biology and Immunology, Virgen Macarena University Hospital, University of Sevilla, Av. Dr. Fedriani 3, 41071-Sevilla, Spain; 3 MD, PhD, Professor, Department of Medical Biochemistry, Molecular Biology and Immunology, Virgen Macarena University Hospital, University of Sevilla, Av. Dr. Fedriani 3, 41071-Sevilla, Spain; 4 MD, PhD, DDS, Professor, Department of Endodontics, School of Dentistry, University of Sevilla, Sevilla, Spain

## Abstract

**Background:**

Leptin, initially described as an adipocyte-derived hormone to regulate weight control, is expressed in normal and inflamed human dental pulp, being up-regulated during pulp experimental inflammation. Leptin receptor (LER) has been identified in human periapical granulomas. The aim of this study was to analyze and characterize the expression of leptin in human periapical granulomas.

**Material and Methods:**

Fifteen periapical inflammatory lesions were obtained from extracted human teeth and teeth which underwent periapical surgery. After their morphological categorization as periapical granulomas and gradation of the inflammatory infiltrate, they were examined by immunohistochemistry using human leptin policlonal antibodies. Leptin mRNA expression was also determined by quantitative real-time PCR (qRT-PCR) and the amount of leptin protein was analyzed by immunoblot.

**Results:**

All periapical lesions exhibited the characteristic of chronic granulomatous inflammatory process with inflammatory infiltrate grade III. Leptin+ cells were detected in 13 periapical granulomas (86.6%). The median number of Leptin+ cells in periapical granulomas was 1.70 (0.00-7.4). Amongst the inflammatory cells in the periapical granulomas, only macrophages were reactive to leptin antibodies. Western blot analysis revealed the presence in all samples of a protein with apparent molecular weight of approximately 16 kDa, corresponding to the estimated molecular weights of leptin. The expression of leptin mRNA was confirmed by qRT-PCR analysis and the size of the amplified fragment (296 bp for leptin and 194 bp for cyclophilin) was assessed by agarose gel electrophoresis.

**Conclusions:**

For the first time, it has been demonstrated that human periapical granuloma expresses the adipokine leptin.

**Key words:** Apical granuloma, dental pulp, endodontics, leptin, leptin receptor, immune system, immunohistochemistry, periapical inflammatory response.

## Introduction

Chronic periapical lesions occur as a result of the immunological response to continuous antigenic stimulation from root canals, and their effects on the systemic health of patient have been investigated (1). The most common periapical lesions are periapical granulomas (2). Periapical granuloma is a chronic inflammatory lesion at the apex of a non-vital tooth consisting of granulation tissue and scar. The inflammatory cell infiltrate in these chronic periapical lesions consists of a mix of T- and B-lymphocytes, polymorphonuclear neutrophils (PMNs), macrophages, dendritic cells (DCs), plasma cells, NK cells, eosinophils, and mast cells, present in different proportions within the granulation tissue of periapical lesions (3). The inflammatory infiltrate constitutes approximately 50% of the cells present in periapical granulomas, with non-inflammatory connective tissue cells, including fibroblasts, vascular endothelium, proliferating epithelium, osteoblasts, and osteoclasts comprising the balance (4). During periapical inflammation, host cells in the periapical tissues release many inflammatory mediators, pro-inflammatory cytokines, and growth factors through innate and adaptive immune responses (3).

Leptin, an adipocyte-derived hormone of 16 kDa regulates weight control (5) but also it has been accepted a role for it regulating immunity, inflammation and hematopoiesis (6).

In fact, it has been classified as a pro-inflammatory cytokine because its primary aminoacid sequence shows structural similarities to the long chain helical cytokine family, such as IL-2, IL-12 and growth hormone (7). Therefore, leptin affects both innate and adaptive immunity exerting an effect on T-cells, monocytes, neutrophils, and endothelial cells (8). Consistent with this role of leptin in the mechanisms of immune response and host defense, leptin levels are increased upon infectious and inflammatory stimuli such as LPS, turpentine, and cytokines (9). Accordingly, leptin receptor (LEPR) shows sequence homology to members of the class I cytokine receptor (gp130) super family (10) and is expressed not only in the central nervous system, but also in hematopoietic and immune systems (11), in mice monocytes and lymphocytes (8,11) and in human peripheral blood T lymphocytes (both CD4 and CD8) (7).

In relation with oral tissues, it has been proposed that leptin can be implicated in inflammatory and local immune responses in human dental pulp (12-14). Moreover, its presence has been reported in healthy and inflamed human dental pulp (13), gingival tissues (15) and in gingival crevicular fluid (16). On the other hand, LEPR protein and LEPR mRNA have been described in healthy and inflamed human dental pulp (14). LEPR gene has been detected in experimental rat periapical lesions (17) and, recently, it has been demonstrated that human periapical granulomas express LEPR (18). However, the expression and immunolocalization of leptin in periapical inflammatory tissues has not been studied. The aim of this study was to analyze and characterize the expression of leptin in human periapical granulomas.

## Material and Methods

The study was carried out with the understanding and written consent of each subject and according to the principles of the World Medical Association Declaration of Helsinki. The protocol was approved by the Ethical Board of the University.

Fifteen human chronic periapical lesions from fifteen healthy, nonsmoking, human donors (45-72 years old), who gave their written informed consent, were obtained from 9 freshly extracted teeth and 6 teeth which undergone periapical surgery. Inflammatory tissues surrounding periapical area were dissected. Each sample was then divided into three parts, one for the Western blotting analysis, other for RNA extraction and quantitative real-time PCR (qRT-PCR) assay, and another one for morphological analysis and immunohistochemistry.

Western blotting analysis was carried out according to the method previously published (18), using horseradish peroxidase-linked anti-mouse/anti rabbit immunoglobulins as secondary antibodies.

Abundance of leptin mRNA was determined by quantitative real-time PCR reaction (qRT-PCR), as described previously (18), using the primers based on the sequences of the National Center for Biotechnology Information GenBank database.

For morphological analysis, the excised periapical lesions were fixed in 10% formalin for at least 24 hours and then embedded in paraffin and processed routinely. A series of 5-μm sections from each tissue sample were cut. Five sections of each serie were stained with haematoxylin-eosin (H&E) to study the histology and to confirm the diagnosis of apical granuloma. The intensity of the inflammatory infiltrate was evaluated according to the method proposed by Peixoto *et al*. (19). Briefly, each specimen was graded at x40 magnification as: grade I, inflammatory cells less than one-third; grade II, inflammatory cells between one third and two-thirds; and grade III, inflammatory cells more than two-thirds.

Another five sections was used for immunohistochemical staining for expression of leptin. Immunohistochemistry technique was carried out as previously described (18), using rabbit antihuman leptin policlonal primary antibody. Human placenta was used as a positive control for leptin expression. Finally, the immunohistochemical specimens were examined using a Leica Laborlux S Microscope (Leica Microsystem GmbH Wetzlar, Germany) with a Nikon DSL2 photo digital system (Nikon Corp, Tokyo, Japan). Each sample was analyzed with a double-blind system by two different operators under magnifications up to x100. A cell was considered as positive when it demonstrated distinct brown surface staining. Six representative sites in each sample were photographed at 1.25, 4, 10, 20, 40, 60 and 100x magnification and captured with a software system (CS3, version 10.0.1; Adobe Photos hop, San Jose, CA, USA). At x20 magnification, five fields with the largest number of immunostained cells were identified. In these fields, leptin+ cells were counted at x40 magnification and the total number of positive cells per specimen was calculated.

The results obtained were submitted to statistical analysis. Since no case of periapical granuloma with inflammatory infiltrate grade I or II was detected in this study, analysis of the number of leptin+ cells according to intensity of the inflammatory infiltrate was not performed. For all tests, significance level was set at 5% (*p* < 0.05). Experiments were repeated separately at least three times to assure reproducible results. Results are expressed as mean ± standard deviation (SD) in arbitrary units (AU). Arbitrary units were calculated as normalized band intensity in Western blot analysis. Statistical analysis was performed using the GraphPad Prism computer program (GraphPad Software, San Diego, CA. USA).

## Results

**Figure 1:**
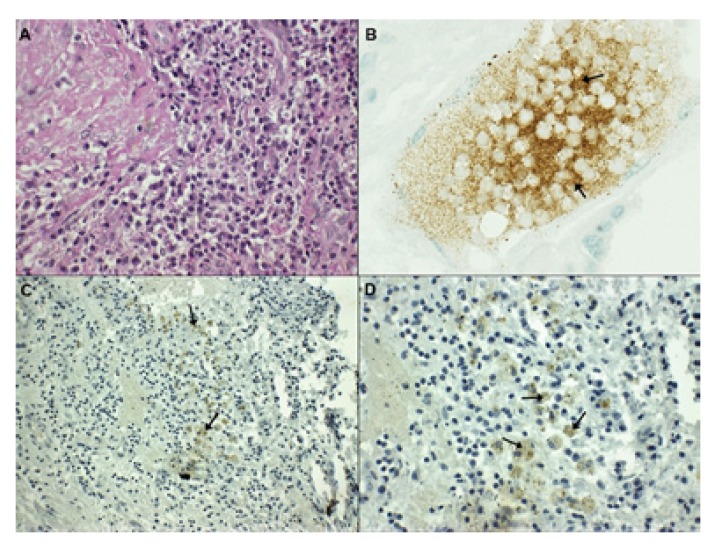
A: Grade III inflammatory infiltrate in periapical granuloma (H&E x40). B: Presence of leptin+ cells in human placenta (positive control, x60). C: Presence of leptin+ cells (arrows) in periapical granuloma (x20). D: Presence of leptin+ cells (arrows) in periapical granuloma (x40).

All periapical lesions examined by light microscopy exhibited a large number of infiltrated inflammatory cells, showing the characteristic of the chronic granulomatous inflammatory process (Fig. 1A): numerous connective tissue fibers, and inflammatory infiltrate constituted by a dense accumulation of round cells (plasma cells and small lymphocytes), macrophages and occasional polymorphonuclear (PMN) leukocytes. No epithelial cells were observed in these samples and all specimens were classified as periapical granulomas. Analysis of the inflammatory infiltrate revealed all cases (n = 15; 100%) with inflammatory infiltrate grade III.

**Figure 2:**
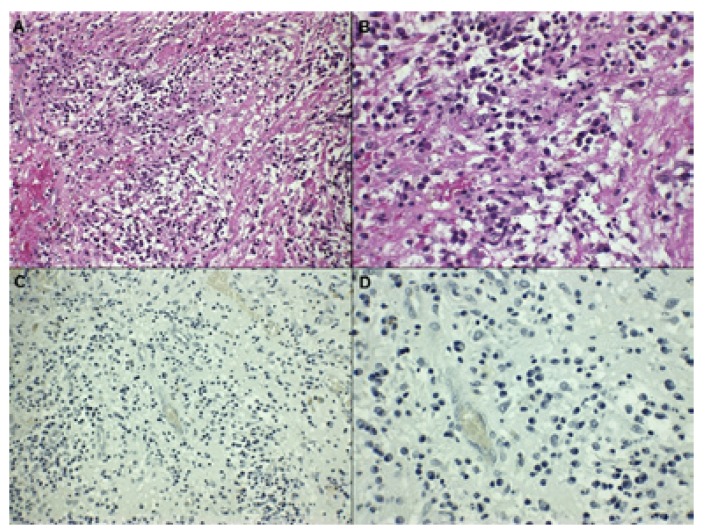
A: Grade III inflammatory infiltrate in periapical granuloma (H&E x20). B: Grade III inflammatory infiltrate in periapical granuloma (H&E x40). C: Absence of leptin+ cells in periapical granuloma (x20). D: Absence of leptin+ cells in periapical granuloma (x40).

The positive control (human placenta) staining pattern resembled that of leptin immunoreactivity (Fig. 1B). Analysis of the immunohistochemical expression of anti-leptin antibody showed the presence of leptin+ cells in 13 periapical granulomas (86.6%). Amongst the inflammatory cells in the periapical granulomas, only macrophages were reactive to leptin antibodies (Fig. 1C, D). Plasma cells, lymphocytes, PMNs, fibroblasts, or endothelial cells did not show expression of leptin. The median number of leptin+ cells in periapical granulomas was 1.70 (0.00 -7.4). Two specimens of periapical granulomas did not show leptin+ cells (Fig. 2).

**Figure 3:**
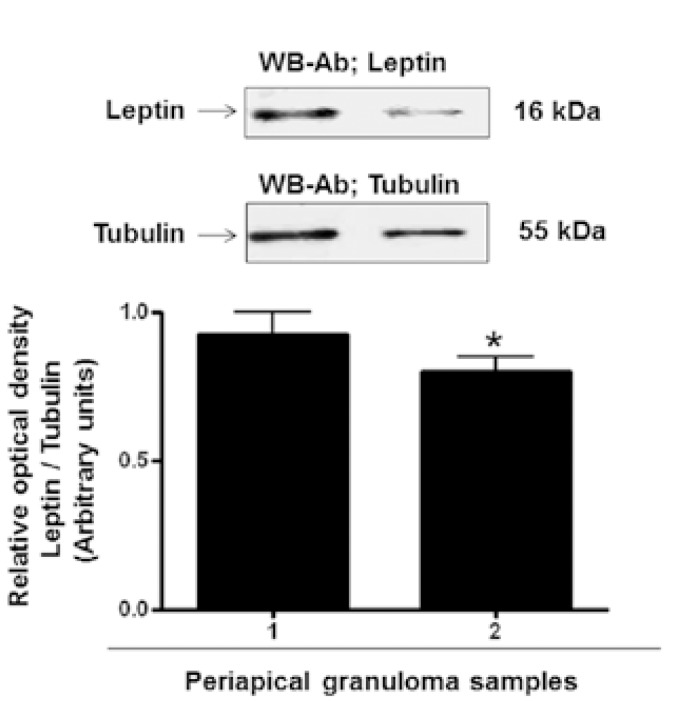
Expression of leptin in human periapical granuloma. To calculate relative amounts of leptin expression, the band intensity of each sample was normalized with tubulin. Densitograms are expressed as means ± standard deviations in arbitrary optical units, calculated as normalized band intensity. Results shown in the immunoblot are from a representative experiment with two dental pulp samples repeated three times.

All human periapical granuloma samples expressed leptin. Western blot analysis revealed the presence in the samples of a protein with apparent molecular weight of 16 kDa, which corresponds to the estimated molecular weight of leptin (Fig. 3).

**Figure 4:**
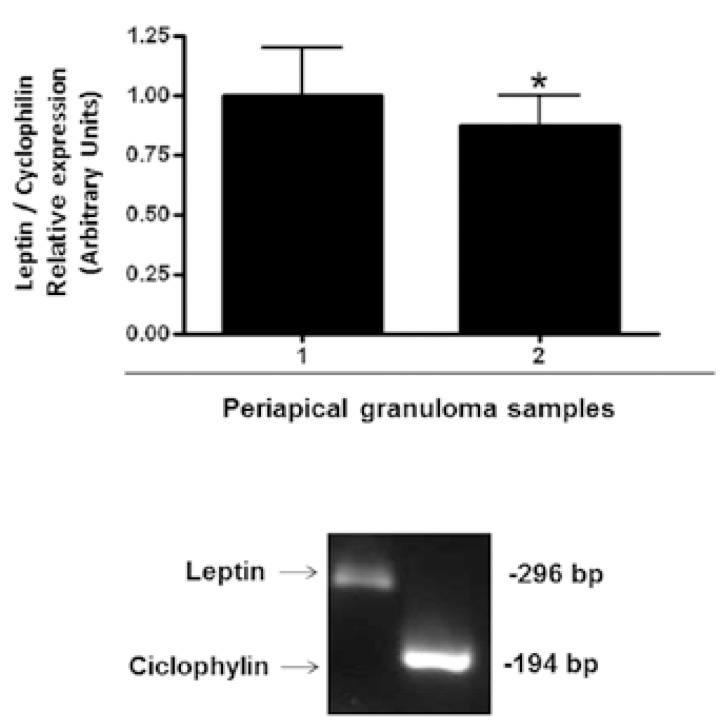
Leptin mRNA expression in human periapical granuloma. Total RNA was extracted from each periapical granuloma sample. Leptin mRNA was quantified using quantitative real time PCR (qRT-PCR) assay, using cyclophilin as internal standard. Results from a representative experiment with two periapical granuloma samples repeated three times are shown. Data represent fold change from the mean values of cyclophilin and are expressed as means ± SD (upper panel). The size of the amplified fragments was confirmed by agarose gel electrophoresis (lower panel).

To further validate the expression of leptin in human periapical granulomas, a real-time PCR assay was performed to examine the messenger RNA (mRNA) levels of leptin in the inflammatory tissues (leptin/cyclophilin ratio: 0.91 ± 0.18) (Fig. 4, upper panel). The size of the amplified fragments (296 bp for leptin and 194 bp for cyclophilin) was confirmed by agarose gel electrophoresis (Fig. 4, lower panel).

## Discussion

This study is the first to demonstrate, both immunohistochemically and at the level of mRNA and protein, the expression of leptin in human periapical granuloma samples.

Leptin is an important hormone secreted by adipose tissue (5). Leptin implication in the control of metabolism and energy homeostasis at central level has been largely described (9). One of these functions is the connection between nutritional status and immune competence (7,9). The adipocyte-derived hormone leptin has been shown to regulate the immune response, innate and adaptive response, both in normal and pathological conditions (6,7). Leptin share helical structure, functions and receptor subunit makeup, with the IL-6 family of cytokines (7). IL-6, IL-11, leukemia inhibitory factor (LIF), oncostatin M (OSM), cardiotrophin-1 (CT-1), ciliary neurotrophic factor (CNTF), and cardiotrophin-like cytokine (CLC) are included in the IL-6 family of cytokines, being all pleiotropic and exhibiting overlapping biological functions (20). Leptin production is increased during infection and inflammation (21), up-regulating both phagocytosis and the production of pro-inflammatory cytokines of the acute-phase response (3,21,22).

In the present study, 13 periapical granulomas (86.6%) were reactive to leptin antibodies. Since analysis of the inflammatory infiltrate revealed 100% of periapical granulomas with inflammatory infiltrate grade III and no case of inflammatory infiltrate grade I or II was detected, analysis of the number of Leptin+ cells according to intensity of the inflammatory infiltrate could not be performed.

The level of leptin mRNA found in periapical granulomas (leptin/cyclophilin ratio: 0.91 ± 0.18) was higher compared to that observed in human pulp samples (leptin/cyclophilin ratio: 0.085 ± 0.015) (13). Earlier studies have shown that human dental pulp express leptin, being increased its expression in the presence of pulp inflammation (13). Taking into account the detection of LEPR in normal and inflamed human dental pulp (14) and in human periapical granulomas (18), together with the immunohistochemical demonstration of leptin expression in human periapical granulomas, showed in the present study, support the role of leptin in periapical inflammatory process. Moreover, previous and present results suggest that leptin expression correlated with the grade of periodontal (16) and pulp (13,14) inflammation.

In human periapical granulomas is characteristic an organization of profuse collagen fibers in diverse directions appearing as irregular dense connective tissue with vascular elements. Inflammatory elements of the connective tissue, such as histio-cytes/macrophages, lymphocytes, neutrophils, eosinophils, and multi nucleated giant cells are present in granulomatous inflammation (5,8). In the present study, the expression of leptin in the inflammatory tissue of periapical granulomas has been demonstrated immunohistochemically. Leptin detected in this tissue can has two origins: circulating leptin or leptin bound to its receptor in immune and endothelial cells. Thus, LEPR has been detected in CD8+ T lymphocytes (23), B lymphocytes (24), human monocyte/macrophages (25), and human neutrophils (26). Accordingly, increased expression of LEPR has been observed in macrophages/foam cells and the endothelial lining of the intima of neo-revascularized regions of human atherosclerotic aorta (27). Since leptin induces endothelial cell proliferation and angiogenesis (27), the presence of leptin in human periapical granulomas suggest a role of leptin in periapical healing.

It has been shown that leptin regulates bone formation and positively promotes ossification through multiple ways, including bone mineralization, remodeling, resorption and osteoblast differentiation (28). Because of this, several studies have assessed the possible implication of leptin in periodontal disease. The presence of leptin has been reported in healthy and inflamed gingival tissues (15) and in gingival crevicular fluid (16). A decrease in serum leptin levels following non-surgical periodontal treatment has been reported (29), which could be associated with a decrease in insulin resistance in the obese population (30).

In conclusion, the expression of leptin in human periapical granulomas demonstrated in the present study, points to a possible role of leptin in periapical immune, inflammatory and reparative responses. Further studies are required analyzing the expression of LEPR in periapical granulomas in order to fully elucidate the roles of leptin and LEPR in the physiology of periapical chronic inflammatory response.
